# A qualitative analysis of third-year medical students’ reflection essays regarding the impact of COVID-19 on their education

**DOI:** 10.1186/s12909-021-02906-2

**Published:** 2021-09-09

**Authors:** Erin L. Kelly, Allison R. Casola, Kelsey Smith, Samantha Kelly, Maria Syl D. de la Cruz

**Affiliations:** Department of Family and Community Medicine, Sidney Kimmel Medical College at Thomas Jefferson University, 1015 Walnut Street, Curtis Building, Suite 400, Philadelphia, PA 19107 USA

**Keywords:** Undergraduate medical education, COVID-19, Workforce development, Clinical training

## Abstract

**Background:**

The COVID-19 pandemic fundamentally changed every aspect of healthcare delivery and training. Few studies have reported on the impact of these changes on the experiences, skill development, and career expectations of medical students.

**Method:**

Using 59 responses to a short reflection essay prompt, 3rd year medical students in Philadelphia described how the COVID-19 pandemic affected their education in mid-2020. Using conventional content analysis, six main themes were identified across 14 codes.

**Results:**

Students reported concerns regarding their decreased clinical skill training and specialty exposure on their career development due to the loss of in-person experience during their family medicine clerkship. A small number felt very let down and exploited by the continued high cost of tuition while missing clinical interactions. However, many students also expressed professional pride and derived meaning from limited patient and mentorship opportunities. Many students developed a new sense of purpose and a call to become stronger public health and patient advocates.

**Conclusions:**

The medical field will need to adapt to support medical students adversely impacted by the COVID-19 pandemic, from an educational and mental health standpoint. However, there are encouraging signs that this may also galvanize many students to engage in leadership roles in their communities, to become more empathetic and thoughtful physicians, and to redesign healthcare in the future to better meet the needs of their most vulnerable patients.

The COVID-19 pandemic significantly disrupted medical education. Globally, many undergraduate medical schools suspended in-person classes and clinical rotations and shifted to asynchronous/synchronous learning platforms and telehealth appointments to keep patients, practitioners, and students safe [6]. This fundamental shift in the structure of medical education has had potential to enormously impact the foundational activities that guide and shape physician skill sets (e.g., in-person patient interaction, observation of physical exam skills, hands-on clinical assessment, bedside teaching). Third-year medical students, in particular, have faced a loss of clinical time and patient interaction in the crucial year of core clinical clerkships [2, 6, 11]. During the third year of medical school, students are exposed to different clinical rotations with the expectation that they will use these experiences to inform their selection of a specialty. Less clinical time translates to decreased exposure to fields of interest, fewer opportunities to hone clinical assessment, and less one-on-one guidance and mentorship.

Several previous COVID-19 studies focused on medical students’ views of the impact of remote learning on their education [6, 10, 11, 13]. Second-year students reported the negative impact that remote learning had on their preparation and training, particularly with regards to the United States Medical Licensing Examination (USMLE) Step 1 examination and learning clinical skills [13]. Clinical educators fear that socially distant learning processes and specialty selection will have long-lasting impacts on students’ skills, empathy, and career development [14]. However, many editorials also illustrate educators’ desires to find a “silver lining” in the chaos, such as the benefit of virtual learning platforms and telehealth, the necessity of integrated public health education, and students’ extraordinary display of adaptability [5, 9, 16]. Perhaps even more concerning, however, is the underexplored loss of skill and career development experienced by third-year medical students during this pivotal period of clinical education. In a recent scoping review conducted by Daniel et al. [4], most undergraduate medical education research focused on surgical students, and none included those in family medicine clerkships [4]. Thus, the goal of the present study is to understand the effects of the COVID-19 pandemic on U.S. third-year medical students’ experiences of education, skill development, and career expectations through qualitative analysis. The third year of undergraduate medical education is truly foundational for clinical skill development and specialty exposure and selection. These students could feel the enduring impacts of COVID-19 in a manner distinctive from their peers. Findings from this work will explore how COVID-19 impacted the daily structure of medical education during this critical juncture.

## Method

### Sample

Participants included two cohorts of third-year medical students completing their Family and Community Medicine clerkship at a large, private medical school in an urban northeastern city in the United States. For a brief period, April 13th, 2020 through June 15th, 2020, all medical students university-wide shifted to completely virtual learning. This institutional change resulted in requiring students to complete an 8-week “Clinical Continuum Course” and a 1-week “Transition to Clerkship” before returning for in-person clinical duties. In-person research and hands-on clinical learning resumed in mid- June with new institutional guidelines designed to benefit student learning while also maintaining safety precautions for COVID-19.

Cohort 1 (*n* = 29) was from June 15th to July 15th 2020 and Cohort 2 (*n* = 30) was from July 20th to August 21st 2020. Cohort 1 (C1: Mean Age = 25.8, *SD* = 1.74; 12 male, 17 female) Cohort 2 (C2: Mean Age = 25.3, *SD* = 1.27; 18 male, 12 female). The race/ethnicities of the 2020–2021 medical student class are 64.3% White, 24.2% Asian, 6.5% Other, 2.5% Black or AA, 2.2% choose not to disclose, 0.4% Pacific Islander. 89.1% are not Hispanic, Latino, Spanish. Five students did not respond for a response rate of 92% (*n* = 59/64).

### Procedures

As part of the Family Medicine Clerkship, all students are required to complete a Quality Improvement and Health Disparities Project. Students complete asynchronous online lessons on the fundamental principles of quality improvement, how to complete a Plan-Do-Study Act cycle (a model for implementing a change process), an introduction to a Community Health Needs Assessment, and a 1-1.5 hour module on health disparities in a vulnerable population of their choice (homeless, veteran, and immigrant/refugee). During their six-week Family Medicine rotation, the students must choose a process that needs improvement in health care and apply their knowledge gained regarding quality improvement and population health. The students provide a visual model of their quality improvement intervention through a process mapping assignment. They are also asked to complete a reflection assignment, which includes a discussion of how their quality improvement intervention will impact vulnerable populations. As part of a larger 10-item short essay reflection assignment that comprehensively evaluates their medical experiences and their perceptions of their patients’ social determinants of health and healthcare, students responded to the prompt: *“Describe how COVID-19 has affected how you view your medical training.”* Essays were between 175 and 250 words and submitted online via CANVAS at the conclusion of the clerkship rotation. The Institutional Review Board of Thomas Jefferson University determined that this study was Exempt from IRB approval and granted a waiver for consent (IRB 2405; Protocol 17E.486). All students practice reflection essay responses starting in the first year of medical school training. In the first year, students are taught to describe their experiences, then evaluate (strengths, areas for improvement, feelings, and assumptions), analyze the impacts on them personally and on the broader field, and to develop action plans, which reflects the main principles of the Integrated Reflection Cycle [1]. Students continue the practice of reflection essays in the second year of medical school, so by their third-year clerkships, they have had significant prior experience with reflection skills and techniques.

### Analysis

**Fig. 1:**
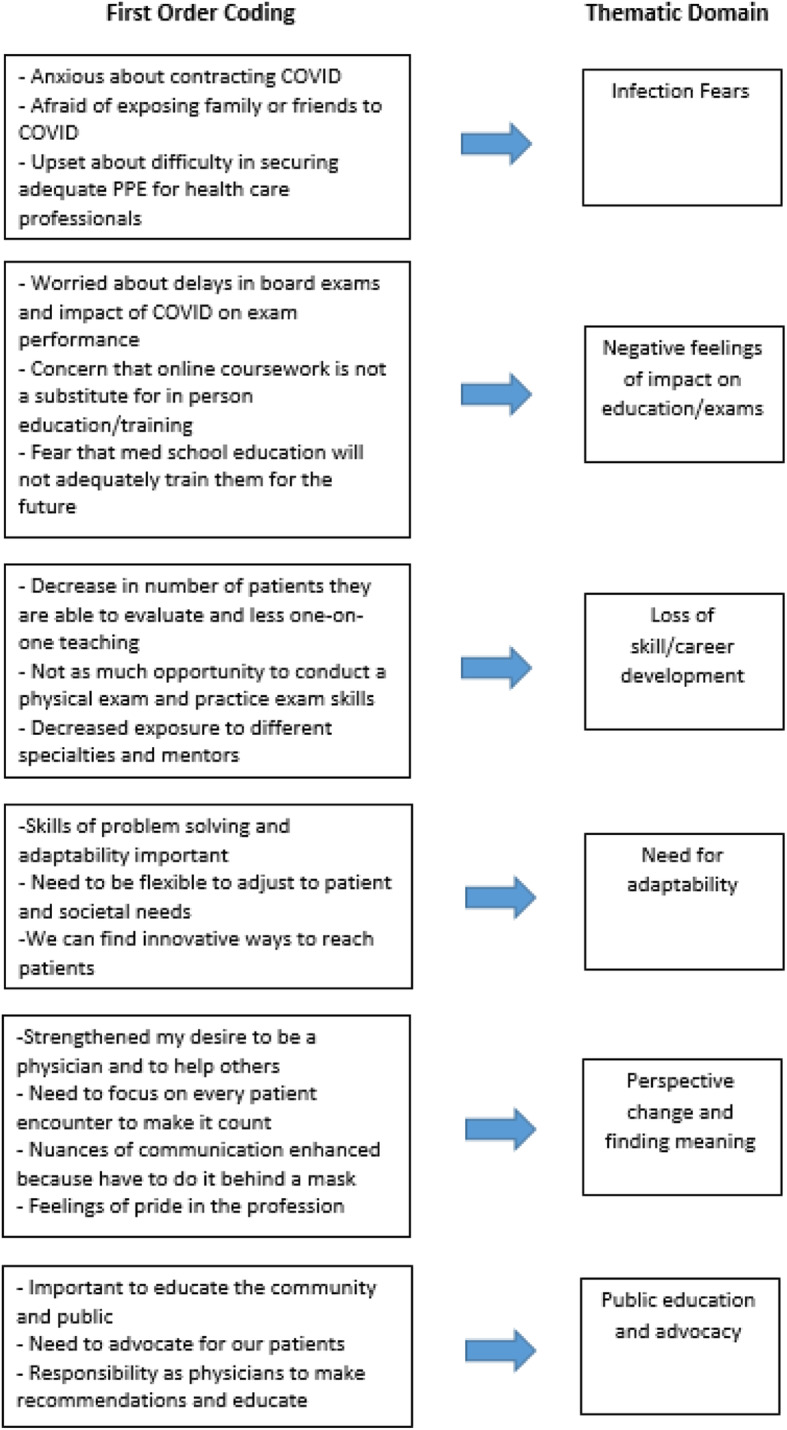
Summary of codebook refinement and final theme determination

Four authors (2 with PhDs, 1 with an MD, and 1 with a BA) reviewed each transcript, highlighted key passages, and made notes of potential themes in the margins to develop the preliminary basis of our codebook per the process recommendations of Huberman and Miles [8]. We developed codes that were derived from the data (open coding) and modified them through consensus before and during our coding process while using a content analysis approach [7]. All the research team members had experience with qualitative study development and coding and had diverse training backgrounds, with degrees in psychology (PhD), public health (PhD), family medicine (MD), and anthropology (BA). After collaboratively coding 10 responses, the codebook was further refined, and 14 codes were used for analysis yielding 6 main themes (see Fig. 1). Two independent coders reviewed each response, and the team reconciled all coding disagreements. Analysis included monitoring of whether there were differences across the cohorts for any domains. A fifth author (with a MA in East Asian Languages and Civilizations assisted with the thematic analysis. NVivo software (version 12) was used to manage and code data. All research team members completed a series of analytic memos of the original 14 codes and synthesized them collaboratively onto the six major themes.

## Results

### Main themes

Overall, while students identified that their education was negatively impacted, didactically and clinically, both short and long-term; many noted that these experiences helped them to reflect on the profession, and their place within it. Six main themes were identified: infection fears, negative feelings of impact on education/exams; loss of skill development; adaptability; perspective change and finding meaning; public education and advocacy. Illustrative quotes are presented in Table 1.

**Table 1:** Illustrative Quotes of the Six Main Themes

Theme	Number of Students	Exemplar Quote
Infection Fears	7	“Additionally, COVID-19 has been very scary for me as a medical student in terms of contracting the illness while treating patients. I am constantly afraid of being exposed to the virus and transmitting the virus to my family and friends unknowingly. There have been exposures to COVID-19 in my medical student class, and as someone who prior to COVID-19, visited family members every other week, I am now thinking twice about going home regularly.” (C2 R14)
Negative feelings of impact on education and exams	28	“The COVID-19 pandemic shutdown began about one week before I was scheduled to be taking my Step 1 board licensure exam, which was cancelled at the time. Since then, my exam was continuously delayed and rescheduled on a short-term basis over the next 4 months, leading to an incredibly stressful extended study period for my boards…Once our medical training restarted, I was actually studying for my Step 1 licensure until halfway through my rotation, which definitively affected and decreased the amount of time I could spend preparing for my day of working with my attending or residents.” (C1 R15) “Our clinical time in our first two rotations has been cut by about 50–75%, with the excuse that “online” coursework is making up for the rest of the training time we would have had. Online coursework is not helpful in acquiring the skills we need to become a physician, and it is more evident than ever that medical education cares more about generating income than training good physicians and doing right by their students.” (C2 R25)
Loss of skill development	35	“On a more specific level to my medical training, I feel like COVID-19 has directly limited the amount of clinical time and exposure I’ve received. At this point, having completed four weeks of clinicals, I feel like I have had the amount of clinical exposure that in pre-COVID-19 times would equal ~ 1–1 and a half weeks. I feel myself growing more accustomed, adept, and less anxious with patients, but ultimately, I feel like my potential is being limited a ton. Ultimately, I really want to just see more and have more time in the clinic. I feel frustrated because I feel like a lot of my preceptors share the same opinion - they feel like I am improving, but at this point, the secret ingredient to becoming a better 3rd year student is simply just getting in the clinic more.” (C1 R14) “I believe that COVID-19 has negatively impacted my medical training. This is primarily due to the decreased number of patient’s being admitted to hospitals or seen in an office at any point in time…The quality of cases I have seen has also decreased due to the inability to see anyone who is COVID-19 positive, even if they are asymptomatic and are in the hospital for something completely unrelated. In all there is a decreased amount of quality teaching time due to the lack of clinical time received in order to accommodate the increased number of students.” (C2 R16)
Adaptability	21	“I am learning to appreciate that a good medical training is not simply knowing the right information and the most up to date algorithms for patient care, but to be able to adapt and problem solve. I have been able to see physicians adapt quickly and efficiently with the sweeping remote nature of the pandemic, which has highlighted the importance of that adaptable and problem-solving spirit for physicians.” (C1 R23) “I have also learned that a huge part of being a good provider is about adaptability. It is true that we can’t see patients in person as much, but this doesn’t mean that we can’t advocate for them. I have advocated for patients simply over the phone and built relationships through phone calls. It takes extra work and leaving your comfort zone, but it is very much possible.” (C1 R12) “Medical training during the time of COVID-19 is a lesson in flexibility. In fact, I would argue that the entirety of 2020 has been a lesson in flexibility. The past 6 months have been unimaginable and unpredictable, and as such, we’ve had to pivot in our daily lives and make major adjustments to education delivery.” (C2 R26)
Perspective change and finding meaning	45	“The very sudden uptick in illness and suffering this country has faced, and the way in which the medical professions have stood up to meet it, has strengthened my resolve to be a physician.” (C1 R26) “With all this said, despite having a much lower quality education as a result, I do value the little bit of education I have received so much more. I try my best to draw every ounce of meaning and learning from each minute talking to a patient or provider. I try to always think of good questions and think deeply about the cases because I know that the number of encounters will be limited.” (C2 R20)
Public education and advocacy	11	“I think there also comes a newfound responsibility to try and dispel myths, appease fear and remind people to behave responsibly. I choose to focus on the fact that many have a deepened appreciation for the sacrifice that doctors make to take care of their patients and am honored that I’m able to be a part of such a noble profession. This pandemic has showed me that there is need for change and that doctors can play an important role in advocating for such.” (C1 R18) “In pandemic scenarios like the one we face today patients are often confused about what to do. Doctors are in the unique position to educate large segments of the population about safety measures and ways to control the spread of disease. I have found that most patients will truly listen and consider what their doctor tells them.” (C2 R7)

### Fear about infection

Interestingly, student concerns about COVID-19 infection were uncommon. Only one student described his/her disappointment in the medical field for failing to provide adequate PPE for staff and anger that healthcare workers were silenced for speaking out about being endangered due to this. A small number from both cohorts discussed their fears of becoming infected with COVID-19 personally, which consequently increased their stress, anxiety, and impaired concentration. Two students expressed concern about potentially spreading the COVID-19 infection to family or friends and how that impacted their decisions to be around their families. While these students detailed their fears only, one student found a way to make this a meaningful learning experience by increasing awareness of their body language, tone of voice, and facial expressions for creating better rapport with patients.

### Negative feelings of impact on education/exams

Many students conveyed frustration, dissatisfaction, and even anger regarding the adverse impact of COVID-19 on their medical school education. Students from both cohorts expressed this dismay as frustration over having their board exams delayed or rushed, losing clinical time with patients, and receiving less one-on-one attention/guidance from instructors. The same students also discussed their concerns about the limits of virtual courses, how larger cohort sizes of rotations adversely impacted their instruction quality (due to students’ rescheduling and delay of clerkships), and the overwhelming fear that their “*medical education is not good enough for [them] to become the doctors that [they] want to be in the future*” (C2 R19). These worries resulted in a few students directly discussing their anger that their tuition price point had remained the same despite the decrease in quality of their training, leaving them feeling exploited and anxious about their futures. Additionally, while many solely focused on detrimental changes seen in their own education, a few identified the larger causes of these changes, like the economic chain of personal protective equipment distribution. Some respondents also identified the impacts of COVID-19 on student expectations and requirements. Two students expressed that balancing rescheduled exams with rotations was extremely stressful, while several others across both cohorts discussed how quickly they had to adapt to shifting to virtual instruction, learning new protocols, and adjusting to a new reality of medical education.

### Loss of skill development

Most students viewed the scarcity of in-person training as detrimental for skill development, leaving some apprehensive about their future careers as physicians. Although the vast majority of students (14 C1, 20 C2) remained positive regarding their experiences, focusing on the safety of themselves and patients, many also noted less face-to-face interaction impaired their ability to build patient rapport. One student noted that*:“The limits on patient contact are unfortunate but necessary for our and our patients’ safety.” (C1 R13).*

Several students (4 C1, 2 C2) expressed dissatisfaction with online education as a substitute for clinical experience, stating *“in all there is a decreased amount of quality teaching time due to the lack of clinical time.” [C2 R16]* This lack of clinical experience and shortened rotations contributes to a decrease in skill development and specialty exposure, creating fear of long-term issues for career advancement as physicians, even if disruptions were short.

*One less week could mean the difference from exposure to a rare surgery that may make you want to join a field of medicine that you were otherwise thinking that you did not want to do.* [C2 R3]

Concerns about their loss of skill development intertwined with their concerns about their future careers. Many students expressed that COVID-19 created negative feelings toward their future, and several students (largely from Cohort 1) expressed concerns about their career development (…“*ultimately, I feel like my potential is being limited a ton*.” [C1 R14]); with most linking this to their own and other students’ lack of clinical training opportunities posing issues for the field long-term. In a few cases, students’ fears regarding their preparation led them to question whether they should remain in medicine.

### Need for adaptability

A little less than half of students noted adaptability as a requirement for the medical field and for students themselves, and this perception did not differ across the cohorts. The greater preponderance of responses focused on individual adaptability while a few acknowledged how both individuals and systems were affected.

*Medical training during the time of COVID-19 is a lesson in flexibility. In fact, I would argue that the entirety of 2020 has been a lesson in flexibility. The past 6 months have been unimaginable and unpredictable, and as such, we've had to pivot in our daily lives and make major adjustments to education delivery. (C2- R26)*

Some described practical lessons learned, such as adapting to novel or dynamic health care settings and becoming flexible and comfortable with uncertainty. Several students remarked on the medical system’s adaptability but noted it in separate ways. For example, two students focused on its ability to adapt quickly and effectively to new protocols and challenges, while three students focused on the adoption of telehealth. A couple of students were proud to see how physicians stepped up in the face of COVID-19 while others described adapting new ways of interacting with patients and developing meaningful connections, with implications for their approach to clinical care in the future.

### Perspective change and finding meaning

Despite the many adverse effects of the pandemic on their education, most students from both cohorts cognitively reframed their experiences to find deeper meaning in their experiences or discussed how their perspectives had changed in positive ways. However, there was considerable range in the students’ areas of focus. Some students reflected on how they will change their approach to training, patients, and medicine, whereas others considered how it reshaped their perspectives on the medical field and the roles of physicians in society.

Primarily, students in Cohort 2 discussed perspective changes regarding their training, including how COVID-19 helped them to develop skills beyond what they would have learned in didactic training --the need to adapt, manage uncertainty, maximize each moment, value skills beyond clinical treatment alone, and face their future in medicine fearlessly.

Some students juxtaposed their appreciation for developing new skills and perspectives against the backdrop of their decreased skill development or time in clinic. As noted by one student,

*COVID-19 has made it harder to meet the quantity of clinical experiences (less face to face time with patients), but I have counterbalanced this by focusing on the quality of my time with patients. *(C2 R11)

A small subset of students found new appreciation for the values of paying attention to their interactions with patients, such as “*I also appreciate how much extra information a physician or student can get about a patient from physically observing affect, posture,* *etc.*” (C2 R2). This manifested as seeing the importance of building patient connections, the benefit of in-person care to foster face-to-face interactions, and the need to pay attention to tone of voice, body language, and general facial expressions to ensure the best patient connection possible.

A smaller number of students also discussed how the pandemic changed their perspective on the need for systematic healthcare changes including greater attention to health disparities and the importance of team-based care in a health system. It also changed some students’ perspectives on the value of public health education, as several students in Cohort 1 commented on the significance of public health perspectives and initiatives.

### Desire to be public educators/advocates

Many students, predominately in Cohort 2, described an urgent need for physicians to educate the public in ways that are more approachable. Four students emphasized the role of physicians as health advocates, as illustrated by one student that “*it is our job as physicians to advocate…with a ferocity*” (C1 R24). These students discussed that medical trainees need to mobilize to influence the community, advocate for healthcare delivery, address social determinants and gain additional resources for vulnerable populations, and to “*think more about how to work past conspiracy theories with patients*” (C2 R28). Another student stated that future generations of physicians have a responsibility to improve health care and need to use quality improvement tools and concepts to accomplish this.

## Discussion

In their reflection essays, the third-year medical students described the significant impacts that COVID-19 had on their clinical education and on their perspectives about the field of medicine. Many students primarily focused on how the COVID-19 pandemic negatively affected their clinical skill training, preparation for exams, specialty exposure, and career development. However, students simultaneously re-conceptualized their roles as learners, providers, educators, and advocates. Most students cognitively reframed their experiences, reevaluating the skills, values, roles, and leadership within the medical field. They described this perspective in terms of a need for adaptability, pride in their profession, and a desire to assume a stronger advocacy role in the future, which reflects considerable resiliency among students as well as potential signs of how these future providers may see their roles in healthcare evolving in the future.

Similar to the few previously reported quantitative studies on these domains [2, 11], our study found that students expressed concerns that a lack of clinical time and exposure would affect their training and future careers. Moreover, many students attributed feelings of frustration, disappointment, and apprehension related to their preparation for USMLE exams, clinical and communication skills, and specialty selection. Some students in our study also reported difficulty balancing the stressors of rescheduled exams with their rotations, as well as having to adapt to new protocols and guidelines. We found that some respondents also expressed anger and feelings of exploitation regarding their tuition fees when the institution was unable to provide the comparable degree of training that it had previously. This is in line with the numerous class action tuition lawsuits nationwide for missed essential components of the curriculum, such as lectures, patient cases, and procedures [17]. These findings could indicate that these future physicians may be a higher risk for increased stress, anxiety, and depression, and that the field will need to monitor physician risk more closely for mental health issues, suicide risk, and burnout as the enduring effects of these experiences continue to affect students. Despite challenges of the COVID-19 pandemic, students in our study tried to find the “silver linings” and reframed their experiences and perspectives in a positive way that reflects the development of their professional identity formation– they identified the benefit of adaptability, pride in their profession, and a desire to become stronger advocates for patients in the future. Students in other studies have described a similar perspective change after having a transformative experience, including an increase in feelings of advocacy, empowerment to create change, and a commitment to action [3, 12, 15]. While there were few differences between the cohorts, students in cohort 2 in particular described paying closer attention to each and every clinical encounter, focusing on quality and not quantity. They appreciated every nuance of communication, verbal and nonverbal, given the difficulty of communicating behind a mask. The students were grateful for the opportunity to do telehealth since it gave them the chance to have clinical “face time” with patients. Similarly, in a previous study, the majority of students who participated in telehealth expressed increased motivation and appreciation for being able to participate in patient care [13]. Despite the deleterious mental health effects noted above, it is possible that students may have developed important coping strategies that will help them to have greater resilience over time. It will be important to monitor in future research if these experiences sensitized students to their patients’ social determinants of health and if patients rate the empathy of these physicians as greater than those in other cohorts.

We found that students recognized how the pandemic not only affected them as individuals, but also the surrounding systems. Students described feeling called to action, and a sense of pride to be in the medical field. They expressed that they could serve a critical purpose as a physician and advocate. This is similar to efforts by students in other parts of the country who have felt called to action – helping with donations for PPE, participating in food drives, calling patients to provide education and follow up care, or delivering food and prescriptions [16]. It will be important to track in the future if these experiences are leading to long-term changes in the roles that healthcare providers have in advocating for individual patients and at a more systemic level through public policy and health care reform. While medical students are trained to recognize the social determinants of health, these experiences in the community may have sensitized these cohorts to engage in strategies to ameliorate them more directly in the future.

### Implications for medical education

Our study is unique in that it focuses qualitatively on the experiences of third year students in their clinical year, and students’ self-reported decrease in clinical skills training has important implications for their futures as residents and physicians. Because of this lack of clinical exposure, educators will need to be innovative for future curricular development, particularly in the clinical years, to find ways to effectively catch students up to the level they need to be prior to internship. Medical educators will need to continue to utilize technology to assist with learning as well as develop reliable assessment tools to determine clinical competency. Because career development was a major concern for students, educators will need to expand opportunities for career counseling, mentorship, and networking. In a climate where students have had less specialty exposure, specialty interest groups will be even more important for students to learn about specialties and meet attendings/residents as role models. Additionally, while many students feel “called” to help, they are looking for guidance on the best way to engage and serve their communities. Amidst the many competing educational priorities, we need to make sure that we are supporting and listening to the needs of our students.

### Limitations and future directions

There are limitations to our study. The findings of this study may not be generalizable because it was conducted in only one medical school and our sample was predominately white. Thus, more diverse medical students will possibly face different concerns or view them differently than our sample**.** The students also had word restrictions on their essay question and were asked to keep it “reflective.” Our question was open-ended, to allow students to explore their thoughts. Lastly, it was not possible to triangulate responses with other data on this sample, though our data is consistent what other studies and news reports have found with medical students in other years of study and specialties, which supports their credibility.

Future directions include additional studies long-term to measure the competencies of students whose clinical time was directly affected by the pandemic. We also know little about how we are supporting the current trainees caught in the crossfire between school, hands-on training, and personal/family stressors. Future efforts should focus on developing standardized assessment to identify clinical gaps and address those gaps, as well as providing effective mentorship and career counseling for these students. Medical educators also need to provide support and carefully monitor the well-being and wellness of students who may still be struggling in the aftermath of the pandemic.

## Conclusion

The COVID-19 pandemic had a profound effect on clinical education for 3rd year medical students. While students reported negative impacts on their education and career development, they also highlighted the positives of learning to adapt, finding meaning in their experiences, and a desire to serve as public health educators and advocates. It will be valuable to see how these students integrate these lessons in their practice once they become independent physicians.

## Data Availability

The datasets generated and/or analysed during the current study are not publicly available due to the data not being cleaned of information that may inadvertently identify participants but are available from the corresponding author on reasonable request.
